# Simultaneous weak measurement of non-commuting observables: a generalized Arthurs-Kelly protocol

**DOI:** 10.1038/s41598-018-33562-0

**Published:** 2018-10-25

**Authors:** Maicol A. Ochoa, Wolfgang Belzig, Abraham Nitzan

**Affiliations:** 1 Department of Chemistry, University of Pennsylvania, Philadelphia, PA 19104 USA; 2 Department of Physics, University of Konstanz, D-78457 Konstanz, Germany; 3 School of Chemistry, Tel Aviv University, Tel Aviv, 69978 Israel

## Abstract

In contrast to a projective quantum measurement, in a weak measurement the system is only weakly perturbed while only partial information on the measured observable is obtained. A simultaneous measurement of non-commuting observables cannot be projective, however the strongest possible such measurement can be defined as providing their values at the smallest uncertainty limit. Starting with the Arthurs and Kelly (AK) protocol for such measurement of position and momentum, we derive a systematic extension to a corresponding weak measurement along three steps: First, a plausible form of the weak measurement operator analogous to the Gaussian Kraus operator, often used to model a weak measurement of a single observable, is obtained by projecting a naïve extension (valid for commuting observable) onto the corresponding Gabor space. Second, we show that the so obtained set of measurement operators satisfies the normalization condition for the probability to obtain given values of the position and momentum in the weak measurement operation, namely that this set constitutes a positive operator valued measure (POVM) in the position-momentum space. Finally, we show that the so-obtained measurement operator corresponds to a generalization of the AK measurement protocol in which the initial detector wavefunctions is suitable broadened.

## Introduction

The possibility of and limitations on simultaneous measurement of non-commuting variables has repeatedly attracted attention of many theorists over the last half century^1–12^, and is attracting renewed attention recently as new techniques for such measurements are manifested^13,14^. Arthurs and Kelly (AK)^1^ have generalized the von-Neumann’s concept of quantum measurement^15^ to describe such simultaneous measurement of position and momentum of a quantum particle by coupling it to two mutually independent detectors set to detect these variables. Obviously, such a measurement cannot determine position and momentum exactly. As explained in the Supplementary Information Appendix A, we find it useful to present these results in a form which is physically different from, but mathematically equivalent to, that of ref.^1^. In this form, the interaction between the measured particle and the detectors

1 $${H}_{{\rm{i}}nt}=K({\hat{p}}_{1}\hat{x}+{\hat{x}}_{2}\hat{p}),$$

is set so as to shift the position of detector 1 and the momentum of detector 2 by amounts that correspond to the particle’s position and momentum. The highest possible accuracy is obtained when the interaction is set to operate between time 0 and *K*^−1^ and is assumed to dominate the evolution during this time, and when the initial wavefunctions of the measured quantum particle and the two detectors are respectively (${{\rm{\Psi }}}_{B}(\bar{x})$) and

2 $${D}_{1}({\bar{x}}_{1})={(\frac{2}{\pi b})}^{1/4}{e}^{-{\bar{x}}_{1}^{2}/b}\,;\,{\tilde{D}}_{2}({\bar{p}}_{2})={(\frac{2b}{\pi })}^{1/4}{e}^{-b{\bar{p}}_{2}^{2}}$$

(the wavefunction of detector 2 is expressed in the momentum representation and *ħ* is taken 1 throughout) where *b* is an arbitrary parameter of dimension [length^2^]. We use the subscripts B and A to denote the wavefunctions before and after measurement, respectively. Barred momentum and position variables are used to denote dimensioned variables similar to those used in AK. Two main results obtained by AK are expressed as follows: First, if projective measurement of the detector states determine the particle position and momentum to be ${\bar{x}}_{m}$ and ${\bar{p}}_{m}$, then the normalized wavefunction after the measurement is given up to a phase by (see Supplementary Information, Appendix A)

3 $${{\rm{\Psi }}}_{A}(\bar{x})={(\frac{1}{\pi b})}^{1/4}{e}^{i{\bar{p}}_{m}\bar{x}}{e}^{-\frac{1}{2b}{(\bar{x}-{\bar{x}}_{m})}^{2}},$$

irrespective of the form of Ψ_*B*_. Second, the joint probability density to find the values ${\bar{x}}_{m}$ and ${\bar{p}}_{m}$ is

4 $$P({\bar{x}}_{m},{\bar{p}}_{m})=\frac{1}{2\sqrt{{\pi }^{3}b}}{|\int d\bar{x}{{\rm{\Psi }}}_{B}(\bar{x}){e}^{-\frac{{(\bar{x}-{\bar{x}}_{m})}^{2}}{2b}}{e}^{-i\bar{x}{\bar{p}}_{m}}|}^{2}.$$

It is important to note that the choice made by AK to set τ ≡ K*t* = 1 implies that the values obtained for the detectors variables ${\bar{x}}_{m}$ and ${\bar{p}}_{m}$ reflect the values of the position and momentum, $\bar{x}$ and $\bar{p}$, of the system itself. This may be contrasted with shorter time measurements, see Eq. (37) below.

As noted by later authors, see, e.g.^2,3^ the post-measurement wavefunction essentially represents a coherent state. To set the formal relationship we transform to dimensionless variables, *x* and *p*, according to

5 $$\bar{x}/\sqrt{2b}\to x,\,\bar{p}\sqrt{b/2}\to p,\,{\rm{\Psi }}(\bar{x})\to {(2b)}^{1/4}{\rm{\Psi }}(x)$$

so that

6 $${{\rm{\Psi }}}_{A}(x)\to {(\frac{2}{\pi })}^{1/4}{e}^{2i{p}_{m}x}{e}^{-{(x-{x}_{m})}^{2}}\,;\,P({x}_{m},{p}_{m})=\sqrt{\frac{2}{{\pi }^{3}}}{|\int dx{{\rm{\Psi }}}_{B}(x){e}^{-{(x-{x}_{m})}^{2}}{e}^{-2ix{p}_{m}}|}^{2}$$

(In the transformation to dimensionless variables, Eq. (5), we have kept the same notation for the wavefunction before and after the needed scaling). We now define the vector *α*_*m*_ and the coherent state $|{\alpha }_{m}\rangle$ by

7 $${\alpha }_{m}=({x}_{m},{p}_{m})$$

(sometimes conveniently represented as a complex number *α*_*m*_ = *α*(*x*_*m*_, *p*_*m*_) = *x*_*m*_ + *ip*_*m*_), and

8 $$\langle x|{\alpha }_{m}\rangle ={(\frac{2}{\pi })}^{1/4}{e}^{2i{p}_{m}x}{e}^{-{(x-{x}_{m})}^{2}},$$

and note that these coherent states are normalized, $\langle \alpha |\alpha \rangle ={\int }_{-\infty }^{\infty }dx{|\langle x|\alpha \rangle |}^{2}=1$ and satisfy the standard closure relationship

9 $${\pi }^{-1}\int {d}^{2}\alpha |\alpha \rangle \langle \alpha |={\pi }^{-1}{\int }_{-\infty }^{\infty }d{x}_{m}{\int }_{-\infty }^{\infty }d{p}_{m}|\alpha \rangle \langle \alpha |=\hat{I}\,({\rm{unit}}\,{\rm{operator}}).$$

We can then cast the maximum accuracy simultaneous measurement of position and momentum that yields the values *x*_*m*_ and *p*_*m*_ (rendered dimensionless as defined above using an arbitrary parameter *b* of dimension [length^2^] that, as seen above, can be related to the initial detectors’ wavefunctions) in terms of the Kraus operator^16^

10 $${\hat{K}}_{{\alpha }_{m}}=\frac{1}{\sqrt{\pi }}|{\alpha }_{m}\rangle \langle {\alpha }_{m}|,$$

which relates the wavefunction after the measurement to that before it by

11 $${{\rm{\Psi }}}_{A}={\hat{K}}_{{\alpha }_{m}}{{\rm{\Psi }}}_{B}$$

and, in view of (9), fulfills the completeness condition

12 $$\int {d}^{2}\alpha \,{\hat{K}}_{\alpha }{\hat{K}}_{\alpha }^{\dagger }=\hat{I}.$$

Since ${\hat{K}}_{\alpha }{\hat{K}}_{\alpha }^{{\mathbb{\dagger }}}$ are positive and satisfy (12) they constitute a positive operator valued measure (POVM).

The measurement described by the operator (10) is not strong (that is, projective) in the usual von Neumann sense (We use the term “strong measurement” to imply projective measurement when applied to a single observable. “Strongest” is used to imply that two non-commuting observables are determined to within their smallest uncertainty limit. Note that these terms do not refer to the strength of system-detector interaction). Indeed, as shown in^1^, Eq. (4) implies that the variances in the measured quantities *x*_*m*_ and *p*_*m*_ satisfy

13 $$\langle \delta {{\hat{\bar{x}}}_{m}}^{2}\rangle ={\langle \delta {\hat{\bar{x}}}^{2}\rangle }_{B}+b/2\,,\,\langle \delta {{\hat{\bar{p}}}_{m}}^{2}\rangle ={\langle \delta {\hat{\bar{p}}}^{2}\rangle }_{B}+1/(2b),$$

or

$$\langle \delta {{\hat{x}}_{m}}^{2}\rangle ={\langle \delta {\hat{x}}^{2}\rangle }_{B}+1/4,\,\langle \delta {{\hat{p}}_{m}}^{2}\rangle ={\langle \delta {\hat{p}}^{2}\rangle }_{B}+1/4,$$

where ${\langle \delta {\hat{O}}^{2}\rangle }_{B}=\langle {{\rm{\Psi }}}_{B}|{\hat{O}}^{2}|{{\rm{\Psi }}}_{B}\rangle -{\langle {{\rm{\Psi }}}_{B}|\hat{O}|{{\rm{\Psi }}}_{B}\rangle }^{2}$. The increased uncertainty is in turn manifested in the position and momentum variances associated with the final wavefunction Ψ_*A*_, Eq. (3), or ${{\rm{\Psi }}}_{A}=|{\alpha }_{m}\rangle$, Eq. (6). Still, this measurement is the strongest possible for the simultaneous determination of position and momentum in the sense that the results adhere to the minimum possible uncertainty.

The most accurate determination of a single observable A corresponds to a projective measurement that yields an eigenvalue *a*_*j*_ of the Hermitian operator $\hat{A}$ and leaves the system in the corresponding eigenfunction *ϕ*_*j*_ with probability ${|\langle {\varphi }_{j}|{{\rm{\Psi }}}_{B}\rangle |}^{2}$ (again, Ψ_*B*_ is the system wavefunction before measurement). Weaker measurements, which result in less drastic effects on the system state at the cost of yielding less information, can be modelled in many ways. A particularly convenient one is described by the Gaussian Kraus operator

14 $${\hat{K}}_{a}^{\lambda }\equiv {(\frac{2\lambda }{\pi })}^{1/4}\exp [\,-\,\lambda {(a-\hat{A})}^{2}],$$

where the real number *a* is the result of the measurement and λ represents the measurement weakness. The operation of ${\hat{K}}_{a}^{\lambda }$ is most easily seen when expressing the initial wavefunction in the basis of eigenstates of $\hat{A}$, ${\Psi }_{B}={\sum }_{j}{c}_{j}{\varphi }_{j}$, with the corresponding eigenvalues *a*_*j*_

15 $${{\rm{\Psi }}}_{A}={\hat{K}}_{a}^{\lambda }{{\rm{\Psi }}}_{B}={(\frac{2\lambda }{\pi })}^{1/4}\sum _{j}{c}_{j}\exp [\,-\,\lambda {(a-{a}_{j})}^{2}]{\varphi }_{j}\,,$$

when *λ* → 0 all results are possible, that is no information is obtained and the (normalized) wavefunction remains intact. When *λ* → ∞ only an eigenvalue can be obtained and the wavefunction is projected onto the corresponding wavefunction. Furthermore, the completeness equation is satisfied:

16 $$\int \,{\rm{d}}{\rm{a}}\,{\hat{K}}_{a}^{\lambda \dagger }{\hat{K}}_{a}^{\lambda }=\sqrt{\frac{2\lambda }{\pi }}\int \,{\rm{d}}a\,\exp [\,-\,2\lambda {(a-\hat{A})}^{2}]=\hat{I}.$$

Finally, while being a mathematical construct, it can be shown by a simplified version of the procedure of ref.^1^ and Appendix A in the Supplementary Information, that the measurement operation (15) reflects a physical measurement in the sense that it describes the outcome of a quantum evolution of a system comprising interacting system and detector.

Coming back to the simultaneous measurement of position and momentum, we have argued above that the measurement operator ${\hat{K}}_{{\alpha }_{m}}$ of Eq. (10), with the measurement result *α*_*m*_ of Eq. (7) representing the obtained position and momentum, corresponds to the strongest simultaneous measurement of these observables. Indeed, the two-observable measurement operator (10) is the closest analog of a projective measurement. Our aim is to construct a systematic protocol for the simultaneous weak measurement of position and momentum, namely a generalization of (14) for such simultaneous measurement. While the availability of such systematic generalization of (10) can be useful in various contexts, our own motivation is to generalize the concept of continuous weak measurement to this case. A consistent description of continuous weak measurement requires a weakness parameter that scales with time. For the measurement of a single observable this is achieved by assuming that *λ* in Eqs (14 and 16) scales as $\bar{\lambda }dt$ (see, e.g., ref.^17^). An extenstion of (10) to a weak measurement situation, characterized by a suitable weakness parameter λ, is a prerequisite for an analogous procedure.

We propose such a construction in Section 2 and confirm that it complies with the general requirements of a Kraus operator. In section 3 we show that the proposed mathematical construction can be realized as an actual physical measurement, and also compare the consequences of weak measurement resulting from fuzzy initial detector states and that are associated with short duration of the detectors-system interaction. Section 4 summarizes our findings and concludes. In a subsequent publication we will apply the procedures developed here to the description of continuous simultaneous measurement of non-commuting observables and to the analysis of the classical limit of a continously observed system.

## A Krauss Operator for Modeling Weak Simultaneous Measurement of Position and Momentum

Here we propose a generalization of the Gaussian Kraus operator (14) to the simultaneous weak measurement of position and momentum. We start with the observation (see Supplementary Information, Appendix B) that the coherent state representation 〈*α*|Ψ〉, where *α* = (*α*_1_,*α*_2_), of a square-integrable function Ψ is a Gabor transform of this function. In the position representation this takes the form

17 $$\langle \alpha |{\rm{\Psi }}\rangle ={(\frac{2}{\pi })}^{1/4}{\int }_{-{\rm{\infty }}}^{{\rm{\infty }}}dx{e}^{-2i{\alpha }_{2}x}{e}^{-{(x-{\alpha }_{1})}^{2}}\langle x|{\rm{\Psi }}\rangle \,.$$

Just as the position and momentum representations describe a system state in terms of distributions that fully specify the values of the position or momentum variables, respectively, the coherent states representation uses an (overcomplete) basis of states characterized by minimum uncertainty of these two observables. Being functions of two variables, coherent states reside in the function space *L*^2^(*R*^2^) - square integrable functions of two variables. However, being images of functions in *L*^2^(*R*), they occupy only a subspace (henceforth denoted G) of the former. We postulate that, starting from a system in a pure state, a simultaneous measurement of position and momentum leaves the system in another pure state in this subspace. Finally, we note (see Supplementary Information Appendix B) that the operator ${\hat{P}}_{G}\equiv {\pi }^{-1}\int {d}^{2}\alpha |\alpha \rangle \langle \alpha |$ is a projection operator onto subspace G.

The implications of these statements can be seen by reformulating the consideration of the strongest possible simultaneous measurement of position and momentum. Suppose that such measurement has yielded the values *x*_*m*_ and *p*_*m*_. If position and momentum were independent variables so that any initial state can be represented as a function of these two variables, Ψ_*B*_(*x*, *p*), in *L*^2^(*R*^2^), then following the measurement the function would collapse to Ψ_*A*_(*x*, *p*) = Ψ_*B*_(*x*_*m*_, *p*_*m*_)*δ*(*x* − *x*_*m*_)*δ*(*p* − *p*_*m*_) (As usual, these forms should be understood as limits of discrete representations obtained by considering a finite discrete lattice in position space when the lattice size and the density of lattice points increase to infinity). This function however is outside subspace G, and we suggest that the strongest possible measurement yields a wavefunction in G that is closest to it, namely ${{\rm{\Psi }}}_{A}={\hat{P}}_{G}{{\rm{\Psi }}}_{B}({x}_{m},{p}_{m})\delta (x-{x}_{m})\delta (p-{p}_{m})$. This leads to

18 $${{\rm{\Psi }}}_{A}={\pi }^{-1}\int dx\int dp|\alpha (x,p)\rangle \langle \alpha (x,p)|\delta (x-{x}_{m})\delta (p-{p}_{m})\rangle {{\rm{\Psi }}}_{B}({x}_{m},{p}_{m})\,.$$

While the form 〈*α*(*x*, *p*)|*δ*(*x* − *x*_*m*_)*δ*(*p* − *p*_*m*_)〉Ψ_*B*_(*x*_*m*_, *p*_*m*_) appears unusual, its meaning is clear: We want here a scalar product between $|\alpha (x,p)\rangle$ and a product of eigenvectors of the position and momentum operators that correspond to eigenvalues *x*_*m*_ and *p*_*m*_, respectively. Since the latter is proportional to *δ*(*x* − *x*_*m*_)*δ*(*p* − *p*_*m*_) the result of the integration is, up to a constant, $|\alpha ({x}_{m},{p}_{m})\rangle$ in agreement with Eqs. (10 and 11). The normalization constant is not determined by (18) because the projection ${\hat{P}}_{G}$ does not necessarily keep normalization, but we know already that $|\alpha ({x}_{m},{p}_{m})\rangle$, Eq. (8), is normalized.

An extension of this procedure may be used to construct an operator for the weak simultaneous measurement of position and momentum that yields *x*_*m*_ and *p*_*m*_ as results. If these variables were independent (with the corresponding operators mutually commuting) and Ψ_*B*_ was an eigenstate of both with eigenvalues *x* and *p*, we could cast the wavefunction following such measurement as a 2-dimensional generalization of Eq. (14), namely the measurement would transform Ψ_*B*_ according to

19 $$\begin{array}{c}{{\rm{\Psi }}}_{B}\to {(\frac{2\lambda }{\pi })}^{\mathrm{1/2}}\exp [-\lambda {({p}_{m}-p)}^{2}-\lambda {({x}_{m}-x)}^{2}]{{\rm{\Psi }}}_{B}\\ \,\,={(\frac{2\lambda }{\pi })}^{\mathrm{1/2}}\exp [-\lambda {|{\alpha }_{m}-\alpha |}^{2}]{{\rm{\Psi }}}_{B}\end{array}$$

where *α* = *α*(*x*, *p*); *α*_*m*_ = *α*(*x*_*m*_, *p*_*m*_). Obviously, we could use different weakness parameters for the two measurements, but one can always rescale variables to get back the form (19). Projecting onto subspace G of *L*^2^(*R*^2^) we now get

20 $${{\rm{\Psi }}}_{A}={\hat{P}}_{G}\exp [-\lambda {|{\alpha }_{m}-\alpha |}^{2}]{{\rm{\Psi }}}_{B}=N\int {d}^{2}\alpha \,\exp [-\lambda {|{\alpha }_{m}-\alpha |}^{2}]|\alpha \rangle \langle \alpha |{{\rm{\Psi }}}_{B}\rangle ,$$

implying the following form of the required operator

21 $${\hat{K}}_{{\alpha }_{m}}^{\lambda }=N\int {d}^{2}\alpha \,\exp [\,-\,\lambda {|{\alpha }_{m}-\alpha |}^{2}]|\alpha \rangle \langle \alpha |\,,$$

where *N* is a normalization constant. In the Supplementary Information Appendix C, we show that with a proper choice of *N* this operator satisfies the pre-requisite normalization condition for a Krauss operator

22 $$\int {d}^{2}{\alpha }_{m}{\hat{K}}_{{\alpha }_{m}}^{\lambda }{\hat{K}}_{{\alpha }_{m}}^{\lambda \dagger }=1;\,\,N=\sqrt{\frac{\lambda (\lambda +2)}{{\pi }^{3}}}\,,$$

thus confirming that the set of positive operators ${\hat{K}}_{{\alpha }_{m}}^{\lambda }{\hat{K}}_{{\alpha }_{m}}^{\lambda \dagger }$ is a POVM. While the measurement operator associated with the AK protocol is essentially a projection on a coherent state, the new Kraus operator, Eq. (21), is a broadened form of this projection, with the broadening affected by a convolution with a 2-dimensional Gaussian function, exp[−λ|α_m_ − α|^2^], with a broadening parameter λ. Mathematically, this is a generalization of the standard Gaussian Kraus operator, which is a convolution of a projective measurement with 1-dimensional Gaussian function, see, e.g.^18^, to the case of two non-commuting observables, here position and momentum. Physically, as in the case of one observable, this generalization corresponds to a measurement protocol whereupon the detector is tailored to cause lesser system disturbance at the cost of obtaining less information on the (here two) non-commuting observables.

To end this Section we note that such a POVM is not unique. Indeed, the set of properly normalized positive operators ${\hat{K}}_{{\alpha }_{m}}^{\lambda }$ themselves also constitute a POVM since (using (21)) ${\int {d}^{2}\alpha }_{m}{\hat{K}}_{{\alpha }_{m}}^{\lambda }={\pi }^{2}N/\lambda$. This implies that, in principle, the operators ${R}_{{\alpha }_{m}}^{\lambda }$ defined by ${({R}_{{\alpha }_{m}}^{\lambda })}^{2}={K}_{{\alpha }_{m}}^{\lambda }$ could also be used as Kraus operators. Both sets are just mathematical constructs of possible measurement processes. Significantly, we show in the following Section that the operators ${K}_{{\alpha }_{m}}^{\lambda }$ define the outcome of an actual measurement as defined by a procedure analogue to that of ref.^1^.

## Realization of Weak Simultaneous Position-Momentum Measurement

The Kraus-type operator (10) was shown in Sec. 1 to represent an Arthurs-Kelly type measurement with the smallest uncertainty 〈*δx*^2^〉〈*δp*^2^〉 in the determined position and momentum. Here we show that its extension (21) to weaker measurements with larger uncertainties can be realized in a similar way that differ from the original AK measurement only by the choice of the initial detectors wavefunctions.

To this end, we consider an arbitrary quantum state |Ψ_*B*_〉 and the resultant state |Ψ_*A*_〉 defined by the Kraus operator ${K}_{{\alpha }_{m}}^{\lambda }$ of Eq. (21)

23 $$|{{\rm{\Psi }}}_{A}\rangle ={K}_{{\alpha }_{m}}^{\lambda }|{{\rm{\Psi }}}_{B}\rangle =N\int \,{d}^{2}{\alpha }_{0}{e}^{-\lambda |{\alpha }_{0}-{\alpha }_{m}{|}^{2}}|{\alpha }_{0}\rangle \langle {\alpha }_{0}|{{\rm{\Psi }}}_{B}\rangle ,$$

where *α*_0_ = (*x*_0_, *p*_0_) and *α*_*m*_ = (*x*_*m*_, *p*_*m*_). Our aim is to show that this state is obtained by a modified version of the AK protocol. Obviously, the resultant state |Ψ_*A*_〉 depends parametrically on the result (*x*_*m*_, *p*_*m*_) of the simultaneous weak measurement. In the position representation, Eq. (23) takes the form

24 $${{\rm{\Psi }}}_{A}(x;\,{x}_{m},{p}_{m})=N\int \,dy\int \,{d}^{2}{\alpha }_{0}{e}^{-\lambda |{\alpha }_{0}-{\alpha }_{m}{|}^{2}}\langle x|{\alpha }_{0}\rangle \langle {\alpha }_{0}|y\rangle {{\rm{\Psi }}}_{B}(y),$$

where we have used Ψ(*x*) = 〈*x*|Ψ〉. Next, using (8) and its complex conjugate, we obtain

25 $${{\rm{\Psi }}}_{A}(x;{x}_{m},{p}_{m})=N{(\frac{2}{\pi })}^{\mathrm{1/2}}\int \,dy\int \,d{x}_{0}\int \,d{p}_{0}{e}^{-\lambda {({x}_{0}-{x}_{m})}^{2}}{e}^{-\lambda {({p}_{0}-{p}_{m})}^{2}}{e}^{-{(x-{x}_{0})}^{2}}{e}^{-{(y-{x}_{0})}^{2}}{e}^{2i{p}_{0}(x-y)}{{\rm{\Psi }}}_{B}(y\mathrm{).}$$

We can bring this expression into a form closer to that established by Arthurs and Kelly^1^ for the system-detectors wavefunction at a time *t* = *K*^−1^, by introducing the change of variables *ω* = *x* − *y* such that

26 $${{\rm{\Psi }}}_{A}(x;{x}_{m},{p}_{m})=N{(\frac{2}{\pi })}^{\mathrm{1/2}}\int \,d\omega \int \,d{x}_{0}\int \,d{p}_{0}{e}^{-\lambda {({x}_{0}-{x}_{m})}^{2}}{e}^{-\lambda {({p}_{0}-{p}_{m})}^{2}}{e}^{-{(x-{x}_{0})}^{2}}{e}^{-{(x-{x}_{0}-\omega )}^{2}}{e}^{2i{p}_{0}\omega }{{\rm{\Psi }}}_{B}(x-\omega ),$$

and integrating with respect to *x*_0_ and *p*_0_. This leads to (using the value of N from Eq. (22))

27 $${{\rm{\Psi }}}_{A}(x;{x}_{m},{p}_{m})=\frac{\sqrt{2}}{\pi }\int \,d\omega {e}^{-2\lambda /(\lambda +2){({x}_{m}-x+\omega \mathrm{/2})}^{2}}{e}^{-[(\lambda +2)/2\lambda ]{\omega }^{2}}{e}^{2i{p}_{m}\omega }{{\rm{\Psi }}}_{B}(x-\omega \mathrm{).}$$

To connect with the procedure outlined in the Supplementary Information, Appendix A, it is convenient to reverse the change of variables defined in Eq. (5). We get

28 $${{\rm{\Psi }}}_{A}(\bar{x};{\bar{x}}_{m},{\bar{p}}_{m})=\frac{1}{\pi \sqrt{2b}}\int \,d\bar{\omega }{e}^{-\lambda /(\lambda +2)\frac{{({\bar{x}}_{m}-\bar{x}+\bar{\omega }\mathrm{/2})}^{2}}{b}}{e}^{-[(\lambda +2)/\lambda ]\frac{{\bar{\omega }}^{2}}{4b}}{e}^{i{\bar{p}}_{m}\bar{\omega }}{{\rm{\Psi }}}_{B}(\bar{x}-\bar{\omega }),$$

which is equivalent to Eq. (43) (for *Kt* = 1) in the Supplementary Information, Appendix A, provided that the following initial detector wavefunctions are used instead of those in Eqs (45) and (46),

29 $$\begin{array}{rcl}{D}_{1}({x}_{1}) & = & {(\frac{2}{\pi {b}_{1}})}^{1/4}{e}^{-\frac{{\bar{x}}_{1}^{2}}{{b}_{1}}};\\ {D}_{2}({x}_{2}) & = & {(\frac{1}{2{b}_{2}\pi })}^{1/4}{e}^{-{\bar{x}}_{2}^{2}/4{b}_{2}}\Rightarrow {\tilde{D}}_{2}({p}_{2})={(\frac{2{b}_{2}}{\pi })}^{1/4}{e}^{-{b}_{2}{\bar{p}}_{2}^{2}}\end{array}$$

with

30 $${b}_{1}=\frac{(\lambda +2)}{\lambda }b,\,{b}_{2}=\frac{\lambda }{\lambda +2}b.$$

Since the measured observables are the position of detector 1 and momentum of detector 2, the relevant wavefunctions are *D*_1_(*x*_1_) and ${\tilde{D}}_{2}({p}_{2})$. In the limit *λ* → ∞ (*b*_1_, *b*_2_ → *b*) this gives the original measurement scheme as described in the Supplementary Information, Appendix A. For small λ the measurement weakness stems from the broadened detector wavefunctions, ${D}_{1}({\bar{x}}_{1}) \sim \exp (\,-\lambda {{\bar{x}}_{1}}^{2}/2b)$ and ${\tilde{D}}_{2}({\bar{p}}_{2}) \sim \exp (\,-\lambda b{{\bar{p}}_{2}}^{2}/2)$. This can be seen explicitly by calculating the variances in the measured position and momentum: As in Eq. (6), the joint probability density for measuring ${\bar{x}}_{m}$ and ${\bar{p}}_{m}$ can be calculated from (28) using $P({\bar{x}}_{m},{\bar{p}}_{m})={\int }_{-\infty }^{\infty }dx{|{{\rm{\Psi }}}_{A}(x;{\bar{x}}_{m},{\bar{p}}_{m})|}^{2}$. We find (Supplementary Information, Appendix D) that the variances in the weakly measured position and momentum are given by

31 $$\langle \delta {\bar{x}}_{m}^{2}\rangle ={\langle \delta {\bar{x}}^{2}\rangle }_{B}+\frac{{b}_{1}}{4}+\frac{{b}_{2}}{4},$$

32 $$\langle \delta {\bar{p}}_{m}^{2}\rangle ={\langle \delta {\bar{p}}^{2}\rangle }_{B}+\frac{1}{4{b}_{1}}+\frac{1}{4{b}_{2}},$$

which leads to Eq. (13) in the limit *λ* → ∞, while for *λ* → 0 it gives $$\langle \delta {\bar{x}}_{m}^{2}\rangle ={\langle \delta {\bar{x}}^{2}\rangle }_{B}+b/2\lambda $$ and $$\langle \delta {\bar{p}}_{m}^{2}\rangle =$$$${\langle \delta {\bar{p}}^{2}\rangle }_{B}+1/(2b\lambda )$$. We have thus shown that the weak measurement defined by the operator (21), (22) corresponds to an AK measurement protocol with initial detector wavefunctions given by Eqs (29 and 30).

The following points should be made concerning these results:

- The different roles of the parametrs *b* and *λ* should be noticed. *b* is a squeezing parameters and could be set to 1 by rescaling *x* and *p* while *λ* controls the actual broadening of the detectors wavefunctions.
- As already noticed in Section I, the choice made by AK to set *τ* ≡ *Kt* = 1 implies that the shifts, ${\bar{x}}_{m}$ and ${\bar{p}}_{m}$, in the position and momentum of detector 1 and 2 respectively correspond to the values of the position and momentum of system itself. The uncertainty in the latter variables, $\bar{x}$ and $\bar{p}$, corresponds to the excess noise, $\langle \delta {\bar{x}}^{2}\rangle =\langle \delta {\bar{x}}_{m}^{2}\rangle -{\langle \delta {\bar{x}}^{2}\rangle }_{B}$ and $\langle \delta {\bar{p}}^{2}\rangle =\langle \delta {\bar{p}}_{m}^{2}\rangle -{\langle \delta {\bar{p}}^{2}\rangle }_{B}$ in the former. In the limit *λ* → 0 this implies the following expressions for the noise in the simultaneously measured system position and momentum
  33 $$\langle \delta {\bar{x}}^{2}\rangle =b/2\lambda ;\,\langle \delta {\bar{p}}^{2}\rangle =1/(2b\lambda )$$
- In the weak measurement limit (*λ* → 0) our measurement protocol can be brought into close agreement with the quantum Bayesian approach^19,20^ under infinitesimal steps. Assuming that broadening is proportional to the interaction time Δ*t* and introducing the Kraus operators defined in ref.^18^ we get

34 $${K}_{x}({x}_{m})={(\frac{2\tilde{\lambda }{\rm{\Delta }}t}{\pi {b}^{2}})}^{1/4}{\int }_{-\,\infty }^{\infty }dx^{\prime} {e}^{-\frac{\tilde{\lambda }{\rm{\Delta }}t{(x^{\prime} -{x}_{m})}^{2}}{{b}^{2}}}|x^{\prime} \rangle \langle x^{\prime} |$$

35 $${K}_{p}({p}_{m})={(\frac{2\tilde{\lambda }{\rm{\Delta }}t{b}^{2}}{\pi })}^{1/4}{\int }_{-\infty }^{\infty }dx^{\prime} {e}^{-\tilde{\lambda }{\rm{\Delta }}t{b}^{2}{(p^{\prime} -{p}_{m})}^{2}}|p^{\prime} \rangle \langle p^{\prime} |$$

for the consecutive weak measurement of position and momentum on the initial state $|{{\rm{\Psi }}}_{B}\rangle$. The resulting wavefunction reads

36 $${{\rm{\Psi }}}_{A}{\rm{^{\prime} }}(x)=\frac{1}{\pi \sqrt{2{b}^{2}}}\int dx{\rm{^{\prime} }}{e}^{-\frac{\mathop{\lambda }\limits^{ \sim }{\rm{\Delta }}t{(x{\rm{^{\prime} }}-{x}_{m})}^{2}}{{b}^{2}}}{e}^{-\frac{{(x-x{\rm{^{\prime} }})}^{2}}{4{b}^{2}\mathop{\lambda }\limits^{ \sim }{\rm{\Delta }}t}}{e}^{i(x-x{\rm{^{\prime} }}){p}_{m}}{{\rm{\Psi }}}_{B}(x{\rm{^{\prime} }}),$$

which upon subtitution of *x* − *x*′ by $\bar{\omega }$, the identification $\lambda /(\lambda +2)\to \tilde{\lambda }{\rm{\Delta }}t\ll 1$ and the additional rescaling *x*′ → *x*′/2 + *x*/2 shows that our protocol, illustrated by Eq. (28), yields the quantum Bayesian result in the *λ* → 0 limit.

To end this discussion, we note that one can also consider the consequence of measurement as expressed by Eq. (43) in the Supplementary Information, in the limit $\tau \equiv Kt\ll 1$ in which the term of order *τ*^2^ can be disregarded. We then have (cf. Supplementary Information, Appendix A, Eq. (52))

37 $${{\rm{\Psi }}}_{A}(\bar{x};{\bar{x}}_{m},{\bar{p}}_{m},\tau )=\sqrt{\frac{2}{\pi }}{e}^{-{({\bar{x}}_{m}-\bar{x}\tau )}^{2}/b}\int d\bar{p}\,{e}^{-i\bar{p}\bar{x}}{\mathop{{\rm{\Psi }}}\limits^{ \sim }}_{B}(\bar{p}){e}^{-b{({\bar{p}}_{m}-\bar{p}\tau )}^{2}},$$

where

38 $${{\rm{\Psi }}}_{B}(x)=\int dp\,{e}^{-ipx}{\mathop{{\rm{\Psi }}}\limits^{ \sim }}_{B}(p).$$

Eq. (37) shows that upon a simultaneous measurement of position and momentum using a short time (*τ* → 0) interaction, the wavefunction is transformed in a way that reflects shifts of the position and momentum of the detectors 1 and 2 by amounts *xτ* and *pτ*, respectively, where *x* and *p* are the position and momentum associated with the system. The implications of this observation on the measurement process will be discussed elsewhere.

## Summary and Conclusions

We have considered together two fundamental quantum mechanical concepts: the simultaneous observation of non-commuting operators, here focusing on the position and momentum of a quantum particle (Note that equivalent operators are quadratures of the radiation field which can be measured under suitable setups^21^), and weak measurements in which the system state is weakly perturbed at the cost of yielding only partial information on the system. Obviously, the strongest possible measurement of two non-commuting observables cannot be projective. In the specific case of position and momentum such measurement can be realized by the AK protocol under which the measurement is affected by coupling the system to two detectors, one responding to the system position and the other to its momentum. The resulting system state is known to be described by a quasi-projection of the initial state onto a coherent state and the measurement is expressed by the operation of the Kraus operator of Eq. (10) where the complex *α* expresses the measured values of the position and momentum variables. In this paper we have derived the corresponding weak measurement operator, Eqs (21 and 22), characterized by a weakness parameter *λ* that extrapolate between the strongest possible measurement (*λ* → ∞) and the vanishing-strength measurement, (*λ* → 0). We have further shown that this weak measurement operator correspond to a generalized AK measurement protocol that uses suitable broadened detectors’ wavefunctions, Eqs (29 and 30).

The concept of weak measurement follows naturally from the observation that a measurement done on a quantum system necessarily affects its state, and the need to control the state change while extracting information from the system. In particular, this concept is essential when designing continuous measurements. Here we have presented and analyzed a weak measurement protocol for simultaneous observation of position and momentum that can be used to formulate a process of continuous weak simultaneous measurement of these observables and to explore the classical limit of such process. These issues will be studied in a subsequent article. It might be interesting to apply our formalism to further developments in quantum metrology ref.^13^, as well in quantum information and communication based on homodyne/heterodyne detection of quadratures of single photons. Refs^22–24^.

## Electronic supplementary material

Supplementary Information

## Data Availability

The authors declare that the data supporting the findings of this study are available within the article.
