# Supplementary material for: Simultaneous weak measurement of non-commuting observables: a generalized Arthurs-Kelly protocol
Source: Sci Rep. 2018 Oct 25;8:15781. doi: 10.1038/s41598-018-33562-0 (PMC6202392; doi:10.1038/s41598-018-33562-0)
Supplement: Supplementary file 1 — Supplementary Information [file 41598_2018_33562_MOESM1_ESM.pdf]

Simultaneous weak measurement of non-commuting observables: a generalized Arthurs-Kelly protocol

Supplementary Information

M. A. Ochoa[1], W. Belzig[2] and A. Nitzan[1,3]

[1]Department of Chemistry, University of Pennsylvania, Philadelphia, PA 9104

[2]Depatment of Physics, University of Konstanz, D-78457 Konstanz, Germany

[3] School of Chemistry, Tel Aviv University, Tel Aviv 69978, Israel

Appendix A

Here we reproduce the Arthurs-Kelly (AK) scheme for the most accurate determination of position and momentum of a quantum particles using two independent detectors. Note that the position and momentum variables appearing in this appendix correspond (by the chosen form of Eqs. (45) and (46) below) to the barred variables $\bar{x}$ and $\bar{p}$ of Section 1. In departure from the language, but not the contents, of AK, we set the interaction between our system and the detectors to affect a position shift in detector 1 and a momentum shift in detector 2, and assume that following the system-detectors interaction projective measurements made to determine the position of detector 1 and the momentum of detector 2. Specifically, the initial state of the system plus detectors is taken to be

$$\phi(x, x_1, x_2, t=0) = \Psi_B(x) D_1(x_1) D_2(x_2), \tag{39}$$

where $\Psi_B(x)$ is the wavefunction of the measured system before the measurement, while $D_1(x_1)$ and $D_2(x_2)$ are the detectors wavefunctions, all in the position representation. The interaction between system and detectors is chosen to be

$$H_{int} = K(\hat{p}_1 \hat{x} + \hat{x}_2 \hat{p}), \tag{40}$$

where $\hat{x}$ and $\hat{p}$ are the system operators representing the position and momentum whose measurement is required, $\hat{p}_1$ is the momentum operator of detector 1 and $\hat{x}_2$ is the position operator of detector 2. Following AK, we assume that the interaction dominates the time evolution during the time t on which it operates so that

$$\phi(x,x_1,x_2,t) = \exp\left(-itH_{\text{int}}\right)\phi(x,x_1,x_2,t=0)$$
$$= e^{-Ktx(\partial/\partial x_1)}e^{-Ktx_2(\partial/\partial x)}e^{(1/2)K^2t^2x_2(\partial/\partial x_1)}\phi(x,x_1,x_2,t=0) \tag{41}$$

The last form on the right is obtained by using the Baker-Campell-Hausdorff formula to disentangle the $\hat{x}$ and $\hat{p} = -i\partial/\partial x$ opeators. Using (39) leads to

$$\phi(x,x_1,x_2,t) = \Psi_B(x - x_2Kt)D_1\left(x_1 - xKt + \frac{1}{2}x_2K^2t^2\right)D_2(x_2) \tag{42}$$

or, in the p_2 representation for detector 2

$$\phi(x,x_1,p_2,t) = \frac{1}{\sqrt{2\pi}}\int dx_2\Psi_B(x - x_2Kt)D_1\left(x_1 - xKt + \frac{1}{2}x_2K^2t^2\right)D_2(x_2)e^{ip_2x_2} \tag{43}$$

Eq. (43) describes the now entangled state of the system and detectors at the end of the period t during which the interaction has been turned on. The actual act of measurement is completed by making projective determinations of the position of detector 1 and momentum of detector 2 immediately following this period. (Note that because of the choice of interaction (40), the results of these measurement directly provide information on the position and momentum of the system-particle). Denoting the results of these measurements x_m and p_m, the system wavefunction following the measurement is given by

$$\Psi_A\left(x;x_mp_m,t\right) = \phi\left(x,x_m,p_m,t\right) \tag{44}$$

while $\left|\Psi_A\left(x;x_mp_m,t\right)\right|^2$ corresponds to the joint probability distribution to obtain x_m and p_m in the measurement *and* to find the system at x. Next, in analogy to AK, we specify to the following detector wavefunctions

$$D_1(x_1) = \left(\frac{2}{\pi b}\right)^{1/4} e^{-x_1^2/b} \quad ; \quad D_2\left(x_2\right) = \left(\frac{1}{2b\pi}\right)^{1/4} e^{-x_2^2/4b} \tag{45}$$

and will also make use of the wavefunction of detector 2 in the momentum representation

$$\tilde{D}_2(p_2) = \left(\frac{2b}{\pi}\right)^{1/4} e^{-bp_2^2} . \tag{46}$$

Furthermore, following AK, we choose $t = K^{-1}$ and make a change of variable, $x_2 \to u = x - x_2$. This leads to

$$\Psi_A(x;x_m,p_m,t=K^{-1})=\frac{1}{\pi\sqrt{2b}}e^{-(x_m-x)^2/2b}e^{ip_m x}\int du\Psi_B(u)e^{-(x_m-u)^2/2b}e^{-ip_m u} \quad (47)$$

and to the joint probability ditribution to obtain x_m and p_m as results of the measurement

$$P(x_m,p_m,t=K^{-1})=\int dx\,|\,\Psi_A(x;x_m,p_m,t=K^{-1})\,|^2=\frac{1}{2\sqrt{\pi^3 b}}\left|\int du\Psi_B(u)e^{-\frac{(x_m-u)^2}{2b}}e^{-iup_m}\right|^2 \quad (48)$$

For completeness we also examine another limit of this scheme, whereupon instead of taking $\tau\equiv Kt=1$ we assume that $\tau \ll 1$ so that the term of order τ^2 in Eq. (43) can be disregarded. This leads to

$$\phi(x,x_1,p_2,\tau)=\frac{1}{\sqrt{2\pi}}D_1\left(x_1-x\tau\right)\int dx_2\Psi_B(x-x_2\tau)D_2(x_2)e^{-ip_2x_2} \quad (49)$$

or, using (44) - (46)

$$\Psi_A(x;x_m,p_m,\tau)=\frac{1}{\pi\sqrt{2b}}e^{-(x_m-x\tau)^2/b}\int du\Psi_B(x-\tau u)e^{-u^2/4b}e^{-ip_m u} \quad (50)$$

This can be put in a more suggestive form by expanding Ψ_B in momentum eigenstates according to

$$\Psi_B(x-\tau u)=\int dp\,e^{-ip(x-\tau u)}\tilde{\Psi}_B\left(p\right) \quad (51)$$

which leads to

$$\Psi_A(x;x_m,p_m,\tau)=\sqrt{\frac{2}{\pi}}e^{-(x_m-x\tau)^2/b}\int dp\,e^{-ipx}\tilde{\Psi}_B\left(p\right)e^{-b(p_m-p\tau)^2} \quad (52)$$

We see that the measurement transforms the wavefunction in a way that reflects shifting the position and momentum of the detectors 1 and 2 by amounts $x\tau$ and $p\tau$ respectively where x and p are the postion and momentum associated with the system-particle.

Appendix B

Here we cast transformation to coherent state representation, $\Psi\rightarrow\pi^{-1}\int d^2\alpha\left|\alpha\right\rangle\left\langle\alpha\,|\,\Psi\right\rangle$ in the language of the Gabor transform. [25] While we use the terms position and momentum for the variables *x* and *p*, the procedure described below regards just their mutual Fourier-transform association $\tilde{f}\left(p\right)=\left(2\pi\right)^{-1/2}\int_{-\infty}^{\infty}dpe^{ipx}f\left(x\right)$ that connects between the position and momentum

representations of a Hilbert-space vector $|f\rangle$. The Gabor transform $f(x)\to F(\alpha_1,\alpha_2)$ was originally introduced by Gabor[25] for the analysis of minimum uncertainty time-frequency signals. In the present context we define it as

$$F(\alpha_1,\alpha_2)=\left(\frac{2}{\pi}\right)^{1/4}\int_{-\infty}^{\infty} dxe^{-2i\alpha_2 x}e^{-(x-\alpha_1)^2}f(x) \tag{53}$$

with the inverse transform being

$$f(x)=\left(\frac{\pi^5}{2}\right)^{1/4}\int_{-\infty}^{\infty} d\alpha_2 e^{2i\alpha_2 x}\int_{-\infty}^{\infty} d\alpha_1 F(\alpha_1,\alpha_2) \tag{54}$$

In the language of coherent states, the transform (53) is just $\langle\alpha\,|\,f\rangle=\int dx\langle\alpha\,|\,x\rangle\langle x\,|\,f\rangle$ where $\alpha=\alpha_1+i\alpha_2$ and the function $\langle x\,|\,\alpha\rangle=\langle\alpha\,|\,x\rangle^*$ is given by Eqs. (7)-(8). (In the corresponding signal analysis literature this function is referred to as a Gabor wavelet). Obviously, merely introducing this language does not by itself constitute a new development, however new insight (applied in Section 2 of the main text) may be obtained from the following observations:

(a) Eqs. (53) and (54) establish a bijective correspondence between the functions $f(x)$ in $L^2(R)$ and the functions of two variables $F(\alpha_1,\alpha_2)$ in space G. This space, henceforth referred to as Gabor space, is an image of the space $L^2(R)$ and as such is a subspace of the space $L^2(R^2)$ - the space of all square integrable functions of two variables.

(b) All relationships defined in $L^2(R)$ have their equivalent in G. In particular the norm $\|f\|=\left(\int dx|f(x)|^2 dx\right)^{1/2}$ is equal to $\|F\|=\left(\pi^{-1}\int d^2\alpha|F(\alpha_1,\alpha_2)|^2\right)^{1/2}$, where $\int d^2\alpha=\int d\alpha_1\int d\alpha_2$, and the scalar product $\langle f\,|\,g\rangle=\int dxf^*(x)g(x)$ can be expressed as $\langle F\,|\,G\rangle=\pi^{-1}\int d^2\alpha F^*(\alpha_1,\alpha_2)G(\alpha_1,\alpha_2)$. Note that these statements correspond to the identity $\langle f\,|\,g\rangle=\pi^{-1}\int d^2\alpha\langle f\,|\,\alpha\rangle\langle\alpha\,|\,g\rangle$ that is familiar in the coherent states literature.

(c) While the operator $P_G=\pi^{-1}\int d^2\alpha|\alpha\rangle\langle\alpha|$ is often regarded as a unit operator, see Eq. (9), it behaves as such only when it operates on functions in G. When operating on a general function

$\Phi(\alpha_1,\alpha_2)$ in $L_2(R^2)$ it projects onto G. By definition the projected function $P_G\Phi$ is that function in subspace G that is closest to Φ, namely $\|\Phi - P_G\Phi\| < \|\Phi - F\|$ where F is any other function in G.

Appendix C

Here we show that with the proper normalization $\hat{K}_\alpha^\lambda$, Eq. (21), satisfies the closure equation characteristic of a Kraus operator,

$$\hat{I} = \int d^2\alpha \hat{K}_\alpha^\lambda \hat{K}_\alpha^{\lambda\dagger} \qquad (55)$$

$$=|N|^2 \frac{\pi}{2\lambda}\int d^2\alpha' \int d^2\alpha'' e^{-\frac{\lambda}{2}|\alpha'-\alpha''|^2} |\alpha'\rangle\langle\alpha'|\alpha''\rangle\langle\alpha''| \qquad (56)$$

To this end, we show that the matrix element $\langle x|...|y\rangle$ of the operator on the right is $C\delta(x-y)$ and determine N so that the constant C is 1. Evaluating this matrix element requires some caution, applying phase factors consistently. Using the standard expression

$$\langle\alpha'|\alpha''\rangle = \exp\left(-\frac{|\alpha'|^2+|\alpha''|^2}{2}+\alpha'^*\alpha''\right) \qquad (57)$$

where $\alpha = (\alpha_1,\alpha_2)$ also requires that Eq. (8) is modified according to

$$\langle x|\alpha\rangle = \left(\frac{2}{\pi}\right)^{1/4} e^{2i\alpha_2\left(x-(1/2)\alpha_1\right)} e^{-(x-\alpha_1)^2} \qquad (58)$$

(It can be shown that the form of Eqs. (57) and (58) are consistent with each other as written. More generally, if we assign a general phase to the coherent state $\alpha = (\mathrm{Re}\alpha \equiv \alpha_1, \mathrm{Im}\alpha \equiv \alpha_2)$ such that $\langle x|\alpha\rangle = (2/\pi)^{1/4} e^{2i\alpha_2(x-k\alpha_1)} e^{-(x-\alpha_1)^2}$, it leads to $\langle\alpha'|\alpha''\rangle = \int dx \langle\alpha'|x\rangle\langle x|\alpha''\rangle =$ $\exp\left\{-\frac{|\alpha'|^2+|\alpha''|^2}{2}+\alpha_1'\alpha_1''+\alpha_2'\alpha_2''-i(\alpha_2'-\alpha_2'')(\alpha_1'+\alpha_1'')+2ik\left(\alpha_1'\alpha_2'-\alpha_1''\alpha_2''\right)\right\}$, which in turn leads to (57) and (58) for the choice $k=1/2$)

Using (56) as an identity operator in $\delta(x-x') = \langle x|\mathbf{\hat{I}}|x\rangle$ then using (57) and (58) and carrying out the straightforward integrations over α' and α'' we find

$$\delta(X-Y)=|N|^2\sqrt{\frac{2}{\pi}}\frac{\pi^{7/2}}{\sqrt{2}\lambda(\lambda+2)}\delta(X-Y) \tag{59}$$

which implies the choice of N given in Eq. (22).

Appendix D

Here we outline the evaluation of the second moments, Eqs. (31) and (32), of the distribution associated with the weakly measured position and momentum. The starting point is the distribution for these measured variables

$$P(\bar{x}_m,\bar{p}_m)=\int_{-\infty}^{\infty}dx\left|\Psi_A(x;\bar{x}_m,\bar{p}_m)\right|^2 \tag{60}$$

Consider first Ψ_A as given by Eq. (28). It follows that

$$\begin{aligned}\langle\bar{x}_m^2\rangle &= \int d\bar{x}\int d\bar{x}_m\int d\bar{p}_m\,\bar{x}_m^2\,|\,\Psi_A(\bar{x};\bar{x}_m,\bar{p}_m)\,|^2 \\ &= \int d\bar{x}\int d\bar{x}_m\int d\bar{p}_m\,\bar{x}_m^2\left|\int dze^{-(\bar{x}_m-\bar{x}/2-z/2)^2/b_1}e^{-(\bar{x}-z)^2/4b_2}e^{-i\bar{p}_m z}\Psi(z)\right|^2,\end{aligned} \tag{61}$$

where we have defined $z=\bar{x}_m-\bar{\omega}$, by considering first the integral with respect to $\bar{p}_m$, so as to find $\int d\bar{p}_m e^{-i\bar{p}_m(z-z')}=2\pi\delta(z-z')$, and then integrate with respect to z'. Next we compute the resulting integrals with respect to $\bar{x}_m$ and $\bar{x}$, in that order, to obtain

$$\int d\bar{x}_m\bar{x}_m^2 e^{-2(\bar{x}_m-x/2-z/2)^2/b_1}=\frac{1}{4}\left[\left(\bar{x}-z\right)^2+b_1\right]\sqrt{\frac{\pi b_1}{2}} \tag{62}$$

$$\frac{1}{4}\int d\bar{x}(\bar{x}-z)^2 e^{-(\bar{x}-z)^2/2b_2}=\left(z^2+\frac{b_2}{4}\right)\sqrt{2\pi b_2}. \tag{63}$$

This leads to the result in Eq. (31) after finding that $\langle\bar{x}_m\rangle=\int dxx\,|\,\Psi_B(x)\,|^2$ using a similar strategy. To calculate $\langle\delta\bar{p}_m^2\rangle$ we first determine $\langle\bar{p}_m^2\rangle=\int d\bar{x}\int d\bar{x}_m\int d\bar{p}_m\,\bar{p}_m^2\,|\,\Psi_A(\bar{x};\bar{x}_m,\bar{p}_m)\,|^2$ by integrating first with respect to $\bar{x}_m$ and then with respect to $\bar{x}$ as follows

$$\int d\bar{x}_m e^{-(\bar{x}_m-\bar{x}/2-z/2)^2/b_1}e^{-(\bar{x}_m-\bar{x}/2-z'/2)^2/b_1}=\sqrt{\frac{b_1\pi}{2}}e^{-(z'-z)^2/8b_1}, \tag{64}$$

$$\int d\bar{x}e^{-(\bar{x}-z)^2/4b_2}e^{-(\bar{x}-z')^2/4b_2}=\sqrt{2\pi b_2}\,e^{-(z'-z)^2/8b_2}. \tag{65}$$

After that, we use the identity

$$-2\pi \frac{\partial^2}{\partial z'^2} \delta(z'-z) = \int d\overline{p}_m \overline{p}_m^2 e^{-i\overline{p}_m (z'-z)}, \qquad (66)$$

to evaluate the remaining integrals by parts. A similar calculation yields $\langle \overline{p}_m \rangle = \langle \overline{p} \rangle$, which leads to the final result, Eq. (32).

References

[25] Gabor, D. Theory of Communication, Part 1. *J. Inst. of Elect. Eng. Part III, Radio and Communication* **93**, 429 (1946)
